# Hospital Costs of Severe Maternal Morbidity Hospitalizations in the United States from 2014 to 2019: A Nationwide Cross-Sectional Study

**DOI:** 10.1055/a-2618-7331

**Published:** 2025-06-11

**Authors:** Mohammad A. Salameh, Megan E. Branda, Bijan J. Borah, Vanessa E. Torbenson

**Affiliations:** 1 Department of Obstetrics and Gynecology, Mayo Clinic, Rochester, Minnesota; 2 Department of Quantitative Health Sciences, Mayo Clinic, Rochester, Minnesota; 3 Department of Health Care Delivery Research, Mayo Clinic, Rochester, Minnesota

**Keywords:** severe maternal morbidity, administrative data, hospitalization cost, health disparities

## Abstract

**Objective:**

The objective of this study was to estimate the average hospitalization cost (AHC) for deliveries affected by severe maternal morbidity (SMM) and analyze trends from 2014 to 2019. The study also aimed to explore cost stratification based on patient, delivery, and hospital characteristics.

**Study Design:**

Using the National Inpatient Sample dataset, all delivery hospitalizations from 2014 to 2019 were identified. Deliveries affected by SMM were determined based on the Centers for Disease Control definition. Deliveries were categorized into three groups: no SMM (nSMM), any SMM (aSMM), and SMM excluding cases with blood transfusion as the only indicator (SMMeBTo). A regression model accounting for survey design and adjusting for variables including age, race/ethnicity, primary payer, income, delivery method, hospital location/teaching status, and hospital region was used to test the trends in incidence. Hospital charges were adjusted using cost-to-charge ratios and presented in 2022 U.S. dollars ($). A regression model adjusting for the same variables was used to assess costs.

**Results:**

From 2014 to 2019, 4,444,957 deliveries were identified, with a weighted estimate of 22,224,775. The rates of aSMM and SMMeBTo were 1.9 and 0.7%, respectively. AHC was $5,218 (95% confidence intervals [CI]: $5,200–5,235) for nSMM, $11,101 (95% CI: $11,038–11,165) for aSMM, and $11,541 (95% CI: $114,330–11,650) for SMMeBTo. Hospitalization costs across all SMM categories rose annually from 2014 to 2017, decreased in 2018, and peaked in 2019. All races had significantly higher costs than non-Hispanic Whites across all SMM categories. SMM costs were higher for cesarean deliveries. The highest cost was in deliveries involving a temporary tracheostomy. Urban teaching hospitals and those in the Northeast had the highest SMM costs.

**Conclusion:**

Deliveries affected by SMM incur significantly higher costs, with these costs increasing over time. Understanding disparities across patient factors, delivery methods, and hospital characteristics can inform interventions aimed at addressing inequities.

**Key Points:**

- Costs of SMM hospitalizations are rising, even after adjusting for inflation.
- The escalating cost burden is disproportionately shouldered by different racial groups.
- Factors in delivery and hospital settings contribute to the variation in cost.

While pregnancy, delivery, and postpartum recovery are normal milestones in the health and lifespan of many individuals, the process and its sequela can be detrimental to some. Severe maternal morbidity (SMM) is defined as unexpected outcomes of labor or delivery that result in major short- or long-term consequences to the health of the birthing person. 1 In addition to the physical, psychological, and financial impact on the individual, these complications also place a considerable burden on the healthcare system.

National rates of SMM have risen in recent years. 2 The higher cost of delivery hospitalizations affected by SMM has been highlighted by studies that relied on limited local cohorts, 3 4 and others based on national databases. 5 6 To our knowledge, however, none have examined national data to track the trends in SMM-related costs over multiple years. Understanding trends in SMM and associated costs is an integral first step in addressing this public healthcare issue. The aim of our study was to use nationwide data sampling all delivery hospitalizations and provide a yearly trend in the average cost of SMM hospitalizations. Additionally, examining the distribution of these costs can help uncover disparities and highlight areas for intervention. Therefore, we also assess costs across subgroups of interest related to patient characteristics including race and primary payer; delivery characteristics namely delivery method and type of SMM; and hospital characteristics, specifically hospital region and hospital location/teaching status.

## Materials and Methods

This cross-sectional study was conducted using data from the Nationwide Inpatient Sample (NIS) from 2014 to 2019. Established as part of the Healthcare Cost and Utilization Project (HCUP), the NIS is the largest database of all-payer hospital inpatient care in the United States. 7 The database is sponsored by the Agency for Healthcare Research and Quality partnering with organizations responsible for state-level data collection and aims to provide estimates of inpatient care in the country. It approximates a 20% sample of all discharges from hospitals in the United States and ensures the sample is representative of the entire country.

The Center for Disease Control (CDC) has a list of 21 indicators ( Supplementary Table S1 , available in the online version only) obtained from discharge data during delivery hospitalizations that are used to identify SMM deliveries. 1 Each indicator has associated codes from the Ninth and Tenth Revision of the International Classification of Diseases (ICD-9 and ICD-10) Diagnosis and Procedure Codes. ICD-9 codes are used for deliveries from 2006 to the third quarter of 2015. ICD-10 codes are used starting in 2016, and the fourth quarter of 2015 is usually excluded as a transition period.

All national delivery hospitalizations of females aged 12 to 55 years were identified. This was done using an enhanced method for identifying obstetric deliveries developed by Kuklina et al which expands on previous definitions and relies on ICD-9 and ICD-10 codes along with Diagnostic Related Groups codes for deliveries. 8 From this cohort, deliveries with SMM were identified using individual patient NIS discharge data which includes corresponding ICD-9 and ICD-10 codes.

Deliveries were divided into those without SMM (no SMM or nSMM), those with at least one of the 21 SMM indicators (any SMM or aSMM), and those with SMM excluding ones where blood transfusion was the only SMM indicator (SMM excluding BT only or SMMeBTo). This is to account for the large percentage of cases where BT was the only SMM indicator which by some frameworks for defining SMM would not be included. 9 Descriptive statistics of the cohorts are reported with a weighted chi-square test statistic to assess for differences between those with aSMM and nSMM.

The cost was calculated by obtaining NIS data on Hospital Charges and multiplying that by cost-to-charge ratios. Cost-to-charge ratios are obtained from HCUP and are determined based on hospital accounting reports from the Centers for Medicare and Medicaid Services. 10 Cost data was absent in a subset and those were excluded from the cost analysis. The cost was adjusted to 2022 U.S. dollars (USD) and presented as average hospitalization cost (AHC). 11 All analyses accounted for the survey weights and stratifications per HCUP recommendations. A regression model for cost, with a log transformation to account for skewed data was modeled. Adjusted findings are presented, and the weighted chi-square test was completed to compare characteristics of deliveries with varying SMM status.

The model adjusted for variables as defined NIS Description of Data Elements. 12 This included maternal age at admission, race/ethnicity as reported by patients and available in hospital records, 13 expected primary payer, and median household income for patient's ZIP code with quartiles indicating the poorest to wealthiest populations. 14 It also adjusted for year of hospitalization, length of hospital stay, transfer status which indicates whether hospitalization was the result of a transfer from a different facility, 15 and delivery method. 16 The model also adjusted for hospital characteristics including hospital bed size, 17 hospital location/teaching status, and hospital region. A metropolitan statistical area based on the core statistical area designation was considered urban, and a nonmetropolitan statistical area was considered rural. 18 A hospital was considered a teaching hospital if it has one or more Accreditation Councils for Graduates. 18 Hospital region was defined by the U.S. Census Bureau. 19 Interaction between SMM and variables that the model was adjusted for were assessed statistically. While an indicator of interaction was found to be statistically significant potentially due to the large sample size, it was considered not clinically significant ( Supplementary Tables S2–S9 , available in the online version only).

Estimates of the average cost of SMM across the years 2014 to 2019 as well as annual costs are presented with 95% confidence intervals (CI). The multivariable model was then used to stratify cost by race, expected primary payer, delivery method, hospital region, and hospital location/teaching status. Estimated costs of deliveries within the 21 indicators for SMM were analyzed with a regression model, accounting for survey weighting and stratification only, and the average costs along with 95% CI are reported. All analysis was generated using SAS software, Version 9.4. 20

NIS datasets are de-identified and publicly available and hence are exempt from Institutional Review Boards review.

## Results

Between 2014 and 2019, a total of 4,444,957 deliveries were identified with a weighted estimate of 22,224,775 deliveries nationwide. Cost data was absent for 60,212 hospitalizations (1.3%).

### Demographics of Study Cohort

Table 1 shows the demographic distribution of hospitalizations with and without SMM. Hospitalizations with aSMM include those with SMMeBTo while nSMM and aSMM are mutually exclusive.

**Table 1:** Demographic data of identified hospitalizations by SMM status

	nSMM		sSMM		SMMeBTo		*p* -Value a
	*n*	%	*n*	%	*n*	%	
Deliveries, weighted numbers	21,810,065	98.1	414,710	1.9	152,380	0.7	
Maternal age, mean (standard deviation), y	28.8 (0.02)		30.3 (0.04)		32.1 (0.06)		<0.001
Race							
Black, non-Hispanic	3,050,574	14.0	91,290	22.0	31,670	20.8	<0.001
White, non-Hispanic	11,042,999	50.6	170,510	41.1	70,665	46.4	
Hispanic	4,252,013	19.5	87,715	21.2	27,170	17.8	
Asian or Pacific Islander, non-Hispanic	1,230,824	5.6	23,205	5.6	7,430	4.9	
Native American, non-Hispanic	151,825	0.7	4,345	1.0	1,610	1.1	
Other, non-Hispanic	959,490	4.4	19,920	4.8	6,650	4.4	
Unknown	1,122,339	5.1	17,725	4.3	7,185	4.7	
Expected primary payer							
Medicaid	9,314,876	42.7	202,665	48.9	67,245	44.2	<0.001
Private	11,098,980	51.0	175,335	42.3	68,045	44.7	
Self-pay	569,770	2.6	12,105	2.9	4,185	2.8	
Other	798,334	3.7	24,085	5.8	12,680	8.3	
Median household income for patient's ZIP code							
0–25th percentile	6,087,807	28.2	141,170	34.5	48,665	32.4	<0.001
26th–50th percentile	5,459,232	25.3	102,085	25.0	38,260	25.5	
51st–75th percentile	5,320,862	24.7	90,500	22.1	34,820	23.2	
76th–100th percentile	4,700,564	21.8	75,145	18.4	28,535	19.0	
Year of hospitalization							
2014	3,677,840	16.9	72,645	17.5	24,630	16.2	<0.001
2015	3,693,335	16.9	71,760	17.3	25,180	16.5	
2016	3,661,291	16.8	64,395	15.5	25,225	16.6	
2017	3,660,511	16.8	67,475	16.3	24,965	16.4	
2018	3,588,464	16.5	69,025	16.6	26,055	17.1	
2019	3,528,624	16.2	69,410	16.7	26,325	17.3	
Length of hospital stay (d)							
1–2	12,649,023	58.0	86,555	20.9	31,040	20.4	<0.001
3	6,260,538	28.7	116,480	28.1	33,340	21.9	
4+	2,900,399	13.3	211,675	51.0	88,000	57.8	
Transfer status							
Not transferred in	21,402,365	98.8	396,085	96.1	142,110	93.9	<0.001
Transferred from the acute facility	139,000	0.6	12,035	2.9	7,200	4.8	
Transferred from other facilities	126,970	0.6	3,980	1.0	1,985	1.3	
Delivery method							
Cesarean	6,599,287	30.2	220,095	53.1	73,630	48.3	<0.001
Vaginal	15,210,778	69.8	194,615	46.9	78,750	51.7	
Hospital bed size							
Small	4,002,695	18.4	65,700	15.8	22,090	14.5	<0.001
Medium	6,727,455	30.8	121,930	29.4	41,490	27.2	
Large	11,079,915	50.8	227,080	54.8	88,800	58.3	
Hospital location/teaching status							
Rural	2,055,370	9.4	37,120	9.0	9,785	6.4	<0.001
Urban nonteaching	4,982,782	22.8	77,180	18.6	26,440	17.4	
Urban teaching	14,771,913	67.7	300,410	72.4	116,155	76.2	
Region							
Northeast	3,516,665	16.1	77,645	18.7	25,915	17.0=	<0.001
Midwest	4,633,012	21.2	74,020	17.8	33,760	22.2	
South	8,619,786	39.5	173,510	41.8	57,785	37.9	
West	5,040,601	23.1	89,535	21.6	34,920	22.9	

Abbreviations: aSMM, any severe maternal morbidity; BT, blood transfusion; nSMM, no severe maternal morbidity; SMMeBTo, severe maternal morbidity excluding blood transfusion only.

a Comparing aSMM with nSMM.

### Cost Trend Throughout the Years 2014 to 2019

**Fig. 1:**
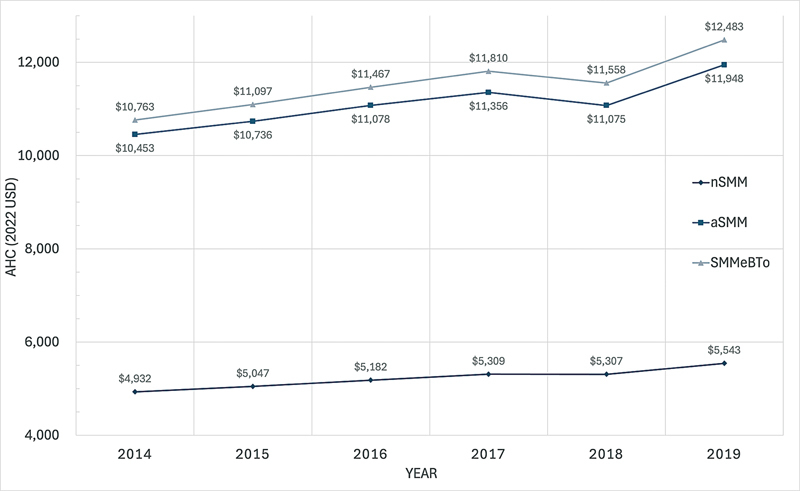
Annual trends in AHC in 2022 USD throughout 2014 to 2019 stratified by severe maternal morbidity status. AHC, average hospitalization cost; aSMM, any severe maternal morbidity; BT, blood transfusion; nSMM, no severe maternal morbidity; SMMeBTo, severe maternal morbidity excluding blood transfusion only; USD, U.S. dollars.

Table 2 and Fig. 1 show the annual AHC from the year 2014 to 2019. SMMeBTo has the highest AHC across the years compared with the other two cohorts (nSMM and aSMM). There is an obvious upward trend in cost over the years after adjusting for inflation with the exception of the year 2018. Between 2014 and 2019, the AHC of nSMM increased by 12.4% ($4,932–5,543) after adjusting for inflation. The AHC of aSMM increased by 14.3% ($10,453–11,948), while the AHC of SMMeBTo increased by 16.0% ($10,763–12,483).

**Table 2:** Annual severe maternal morbidity counts and AHC

Year	nSMM	sSMM	SMMeBTo
2014, weighted *n* (%)	3,677,840 (98.1%)	72,645 (1.9%)	24,630 (0.7%)
Unadjusted AHC (95% CI) a	$4,745 ($4,739–4,750)	$9,694 ($9,581–9,809)	$9,403 ($9,193–9,617)
Adjusted AHC (95% CI) b	$4,932 ($4,916–4,949)	$10,453 ($10,394–10,514)	$10,763 ($10,662–10,864)
2015, weighted *n* (%)	3,693,335 (98.1%)	71,760 (1.9%)	25,180 (0.7%)
Unadjusted AHC (95% CI)	$4,853 ($4,848–4,859)	$9,999 ($9,882–10,117)	$10,050 ($9,821–10,286)
Adjusted AHC (95% CI)	$5,047 ($5,030–5,064)	$10,736 ($10,674–10,798)	$11,097 ($10,993–11,202)
2016, weighted *n* (%)	3,661,291 (98.3%)	64,395 (1.7%)	25,225 (0.7%)
Unadjusted AHC (95% CI)	$4,980 ($4,974–4,986)	$10,537 ($10,408–10,668)	$10,995 ($10,755–11,240)
Adjusted AHC (95% CI)	$5,182 ($5,164–519,9)	$11,078 ($11,014–11,142)	$11,467 ($11,360–11,576)
2017, weighted *n* (%)	3,660,511 (98.2%)	67,475 (1.8%)	24,965 (0.7%)
Unadjusted AHC (95% CI)	$5,100 ($5,094–5,106)	$11,054 ($10,921–11,189)	$11,820 ($11,559–12,087)
Adjusted AHC (95% CI)	$5,309 ($5,291–5,327)	$11,356 ($11,291–11,422)	$11,810 ($11,700–11,922)
2018, weighted *n* (%)	3,588,464 (98.1%)	69,025 (1.9%)	26,055 (0.7%)
Unadjusted AHC (95% CI)	$5,114 ($5,108–5,120)	$11,133 ($11,000–11,267)	$11,922 ($11,660–12,190)
Adjusted AHC (95% CI)	$5,307 ($5,289–5,325)	$11,075 ($11,011–11,139)	$11,558 ($11,449–11,667)
2019, weighted *n* (%)	3,528,624 (98.1%)	69,410 (1.9%)	26,325 (0.7%)
Unadjusted AHC (95% CI)	$5,324 ($5,318–45,331)	$11,622 ($11,485–11,760)	$12,396 ($12,137–12,662)
Adjusted AHC (95% CI)	$5,543 ($5,524–5,562)	$11,948 ($11,879–12,017)	$12,483 ($12,366–12,601)
Averaged over 2014–2019, weighted *n* (%)	21,810,065 (98.1%)	414,710 (1.9%)	152,380 (0.7%)
Unadjusted AHC (95% CI)	$5,013 ($5,011–5,016)	$10,639 ($10,587–10,692)	$11,064 ($10,964–11,166)
Adjusted AHC (95% CI)	$5,218 ($5,200–5,235)	$11,101 ($11,038–11,165)	$11,541 ($11,433–11,650)

Abbreviations: AHC, average hospitalization cost; aSMM, any severe maternal morbidity; BT, blood transfusion; CI, confidence interval; nSMM, no severe maternal morbidity; SMMeBTo, severe maternal morbidity excluding blood transfusion only; USD, U.S. dollars.

a AHC in 2022 USD accounting only for the stratification and weights associated with survey design.

b AHC in 2022 USD adjusted by multivariable model.

**Fig. 2:**
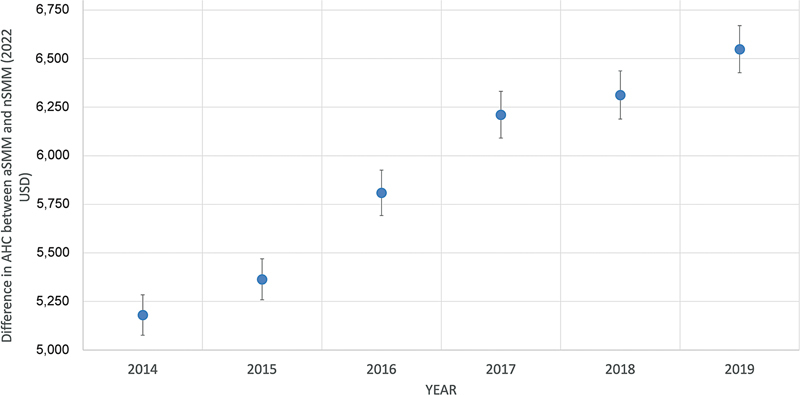
Annual difference in AHC in 2022 USD between aSMM and nSMM from 2014 to 2019. AHC, average hospitalization cost; aSMM, any severe maternal morbidity; nSMM, no severe maternal morbidity; USD, U.S. dollars.

**Fig. 3:**
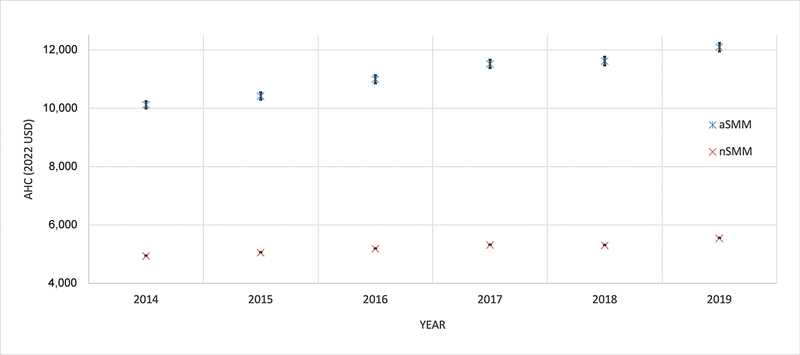
Interaction term between year and SMM. AHC, average hospitalization cost; aSMM, any severe maternal morbidity; nSMM, no severe maternal morbidity; USD, U.S. dollars.

The difference in AHC between nSMM and aSMM increased annually and the difference was significantly higher in 2019 compared with 2014 as seen in Fig. 2 . This is further demonstrated in Fig. 3 displaying the interaction term between SMM and year.

### Cost Stratification by Patient Characteristics

Table 3 shows non-Hispanic White race has the lowest AHC across all SMM status categories. Asian or Pacific Islanders have the highest AHC. Private insurance patients had higher AHC than Medicaid patients with overlap in 95% CI.

**Table 3:** AHC stratified by race and expected primary payer

	AHC (95% CI) a		
	nSMM	aSMM	SMMeBTo
Race			
Black, non-Hispanic	$5,355 ($5,337–5,372)	$11,495 ($11,430–11,560)	$11,871 ($11,760–11,983)
White, non-Hispanic	$5,061 ($5,044–5,078)	$10,556 ($10,496–10,617)	$10,998 ($10,896–11,102)
Hispanic	$5,286 ($5,269–5,304)	$11,228 ($11,165–11,292)	$11,860 ($11,749–11,971)
Asian or Pacific Islander, non-Hispanic	$5,984 ($5,961–6,007)	$12,816 ($12,739–12,894)	$13,636 ($13,506–13,767)
Native American, non-Hispanic	$5,579 ($5,542–5,616)	$11,677 ($11,584–11,772)	$12,317 ($12,183–12,453)
Other, non-Hispanic	$5,331 ($5,311–5,352)	$11,505 ($11,436–11,575)	$12,023 ($11,908–12,139)
Unknown	$5,143 ($5,124–5,162)	$10,866 ($10,802–10,931)	$11,441 ($11,332–11,551)
Expected primary payer			
Medicaid	$5,169 ($5,152–5,186)	$11,164 ($11,101–11,227)	$11,671 ($11,563–11,781)
Private	$5,287 ($5,269–5,306)	$11,258 ($11,193–11,324)	$11,741 ($11,631–11,852)
Self-pay	$4,718 ($4,697–4,739)	$9,510 ($9,450–9,571)	$9,509 ($9,416–9,602)
Other	$5,151 ($5,130–5,173)	$10,202 ($10,139–10,266)	$10,425 ($10,325–10,526)

Abbreviations: AHC, average hospitalization cost; aSMM, any severe maternal morbidity; BT, blood transfusion; CI, confidence interval; nSMM, no severe maternal morbidity; SMMeBTo, severe maternal morbidity excluding blood transfusion only; USD, U.S. dollars.

a Adjusted AHC in 2022 USD averaged over 2014 to 2019.

### Cost Stratification by Delivery Characteristics

Table 4 shows higher AHC across all SMM categories in cesarean deliveries.

**Table 4:** AHC stratified by delivery method

Delivery method	AHC (95% CI) a		
	nSMM	aSMM	SMMeBTo
Vaginal	$4,368 ($4,353–4,383)	$8,292 ($8,244–8,340)	$8,820 ($8,737–8,904)
Cesarean	$7,177 ($7,153–7,202)	$13,582 ($13,505–13,660)	$14,446 ($14,311–14,583)

Abbreviations: AHC, average hospitalization cost; aSMM, any severe maternal morbidity; BT, blood transfusion; CI, confidence interval; nSMM, no severe maternal morbidity; SMMeBTo, severe maternal morbidity excluding blood transfusion only; USD, U.S. dollars.

a Adjusted AHC in 2022 USD averaged over 2014 to 2019.

Table 5 shows the average AHC in deliveries stratified by type of SMM in order of decreasing cost. It is important to note that a delivery might have more than one SMM. For example, deliveries with disseminated intravascular coagulation might have been complicated by hysterectomy and blood transfusion.

**Table 5:** AHC stratified type of SMM affecting the delivery

Type of SMM	AHC (95% CI) a
Temporary tracheostomy	$137,585 ($115,210–164,305)
Ventilation	$31,293 ($30,258–32,363)
Cardiac arrest or ventricular fibrillation	$27,289 ($24,769–30,064)
Acute respiratory distress syndrome	$26,175 ($25,546–26,820)
Shock	$24,080 ($23,331–24,853)
Conversion of cardiac rhythm	$22,634 ($20,505–24,983)
Amniotic fluid embolism	$22,096 ($19,380–25,193)
Hysterectomy	$21,401 ($20,948–21,865)
Acute myocardial infarction	$20,443 ($18,211–22,949)
Sepsis	$18,939 ($18,475–19,414)
Acute renal failure	$15,086 ($14,815–15,362)
Pulmonary edema/acute heart failure	$14,963 ($14,566–15,370)
Sickle cell disease with crisis	$14,929 ($13,995–15,926)
Heart failure/arrest during surgery or procedure	$13,935 ($11,859–16,376)
Air and thrombotic embolism	$13,852 ($13,139–14,602)
Puerperal cerebrovascular disorders	$12,551 ($11,974–13,156)
Aneurysm	$11,057 ($9,621–12,709)
Blood transfusion	$10,400 ($10,342–10,459)
Disseminated intravascular coagulation	$10,064 ($9,890–10,242)
Severe anesthesia complications	$9,610 ($8,943–10,328)
Eclampsia	$9,404 ($9,197–9,615)

Abbreviations: AHC, average hospitalization cost; CI, confidence interval; SMM, severe maternal morbidity; USD, U.S. dollars.

a AHC in 2022 USD averaged over 2014 to 2019.

### Cost Stratification by Hospital Characteristics

Table 6 shows AHC stratified by hospital characteristics. Urban teaching hospitals have the highest cost as do those in the Northeast.

**Table 6:** AHC stratified by hospital location/teaching status

	AHC (95% CI) a		
	nSMM	aSMM	SMMeBTo
Hospital location/teaching status			
Rural	$5,337 ($5,319–5,356)	$11,119 ($11,055–11,184)	$11,613 ($11,504–11,723)
Urban nonteaching	$4,895 ($4,879–4,912)	$10,165 ($10,106–10,224)	$10,529 ($10,430–10,628)
Urban teaching	$5,310 ($5,292–5,328)	$11,340 ($11,275–11,406)	$11,766 ($11,656–11,877)
Hospital region			
Midwest	$5,108 ($5,091–5,126)	$10,910 ($10,847–10,973)	$11,315 ($11,209–11,422)
Northeast	$6,082 ($6,061–6,104)	$13,038 ($12,963–13,114)	$13,366 ($13,241–13,493)
South	$4,527 ($4,512–4,542)	$9,574 ($9,520–9,629)	$9,845 ($9,753–9,938)
West	$5,902 ($5,882–5,923)	$12,562 ($12,489–12,635)	$13,219 ($13,095–13,345)

Abbreviations: AHC, average hospitalization cost; aSMM, any severe maternal morbidity; BT, blood transfusion; CI, confidence interval; nSMM, no severe maternal morbidity; SMMeBTo, severe maternal morbidity excluding blood transfusion only; USD, U.S. dollars.

a Adjusted AHC in 2022 USD averaged over 2014 to 2019.

## Discussion

Our study showed more than doubling of cost in deliveries with SMM compared with those without SMM, and those deliveries with SMM that excluded blood transfusion only had an even higher cost. There was a general increase in the cost of delivery hospitalizations throughout the years after adjusting for inflation and that increase was higher in those affected by SMM. The study investigated the differential distribution of this cost burden showing higher costs in racial minorities. Hospitalizations covered by private insurance had higher AHCs than those covered by Medicaid. Deliveries by cesarean section had higher AHC. There were cost differences across regions and hospital types with SMM deliveries being most expensive in the Northeast and when occurring in urban teaching hospitals.

The higher cost associated with SMM compared with unaffected deliveries is well established, 5 as is the rise in SMM delivery rates over time. 21 22 In addition to reinforcing these points, our study highlighted a progressive increase in average annual costs suggesting a growing scale of the issue. While some highlight the increasing maternal age as a factor in the rise of SMM, 22 several studies have pointed to the growing prevalence of maternal comorbidities as a key factor. 23 24 Our study showed a decrease in AHC in 2018 which may be related to more widespread implementation of accountable care organizations. 25

Racial disparities in the occurrence of SMM are unfortunately widely described. For example, the study by Fingar et al that used the NIS data and the CDC's definition showed 112% higher rates of SMM in Black compared with White patients. 2 Another study showed similarly higher rates of SMM amongst Native Americans. 26 Our study shows an additional layer which is an increase in the average cost of SMM deliveries in those marginalized groups. This means that not only are they more likely to experience SMM, but SMM tends to incur higher costs. All racial groups had significantly higher AHC compared with the White race. Importantly, this was after adjusting for potential confounding factors including the patient's age, primary payer, and median household income of the patient's ZIP code. The racial differences in cost were consistent across all SMM categories.

Disproportionate rates of comorbidities such as chronic hypertension can partially explain the disparities observed in maternal health outcomes, particularly with SMM. 27 However, it is important to understand that these comorbidities are often interlinked with systemic factors. Communities of color face structural barriers such as a lack of affordable insurance and inadequate access to prenatal care. These barriers, often exacerbated by poverty and socioeconomic instability, are not a consequence of individual health behaviors but are deeply rooted in historical and institutional inequities within the healthcare system. 28 29

Our study also highlights disparities affecting an often-overlooked group: Asians and Pacific Islanders. Notably, this group had the highest SMM-associated costs. A study by Siddiqui et al, using NIS data from 2002 to 2013, similarly found higher rates of SMM in this cohort compared with Caucasians. 30 Biological differences including variations in thrombogenic profiles and genetic variation in uterine contractility receptors were been proposed contributors. 31 32 However, it is important to note that Asian or Pacific Islanders in this group had lower comorbidity index and higher income which are thought to be protective. These findings reinforce the hypothesis that systemic biases and cultural influences in healthcare contribute to inequities in maternal outcomes.

Disparities in SMM also extend to differences by expected primary payers. Several studies have shown that Medicaid recipients constitute a relatively higher percentage of deliveries affected by SMM compared with private insurance. 2 4 In our cost analysis, Medicaid recipients had lower AHC with some overlap possibly due to the lower reimbursement rates for Medicaid-covered deliveries. 33 Other studies have shown that while absolute cost is lower in Medicaid patients, SMM causes larger additional costs in Medicaid recipients compared with those with private insurance. For example, in a 2016 retrospective study by Black et al of a large database of insurance claims, SMM caused a 100% additional cost to delivery in Medicaid recipients compared with 59% in those with private insurance. 34 In our study, aSMM caused a 116% additional cost in Medicaid recipients compared with 113% in those with private insurance.

Furthermore, the study showed differences in cost by characteristics related to delivery namely delivery method and type of SMM involved. In this cohort, AHC was higher in cesarean deliveries which literature corroborates. 35 The increase in cost was similar across all SMM status categories—approximately 64% higher in cesarean deliveries. On the other hand, in the study by Vesco et al, the gap between the cost of cesarean deliveries and vaginal deliveries was higher in SMM deliveries. 6 For example, in patients with private insurance, cesarean deliveries without SMM were 41% more expensive than vaginal deliveries without SMM, and cesarean deliveries with SMM were 75% more expensive than vaginal deliveries with SMM. This highlights a potential synergy between SMM and cesarean deliveries.

The highest AHC was in deliveries affected by temporary tracheostomy averaging 26 times the cost of deliveries with nSMM. This can be attributed to the multiple coexisting SMMs that are expected with that procedure. In fact, in the study by Chen et al, deliveries affected by temporary tracheostomy had an average of 6.2 other SMM indicators. 5

Lastly, on a hospital level, our study showed that in deliveries with SMM, the highest AHC was in those performed in urban teaching hospitals. This seems incongruent with NIS data previously published that showed the increased cost of SMM hospitalizations between 2012 and 2014 in rural hospitals relative to urban ones. 36 This could be the result of this study stratifying urban hospitals into teaching and nonteaching hospitals with the higher cost incurred by teaching urban hospitals. 37 Patients with more comorbidities or more complex SMM indicators may be more likely to establish care or be transferred to such hospitals.

Geographically, the highest cost was in those in Northeast hospitals followed by those in the West. Hospitals in the South consistently had the lowest AHC regardless of SMM status. Variation in cost of deliveries by hospital region has been demonstrated in literature with a study that used NIS data showing similarly highest cost in deliveries in the Northeast followed by the West regardless of SMM status. 34

Interventions aimed at preventing SMM and reducing racial gaps should be prioritized. This includes patient, provider, system, and community-level interventions.

Enhancing individual patient access to reproductive services, including contraception, abortion, and preconception counseling improves outcomes. 38 39 Cultural humility training as well as improved diversity representation in healthcare workers are examples of provider-level interventions that can reduce SMM attributable to racism in healthcare. 40 System-level interventions include evidence-based bundles and protocols aimed at reducing SMM which have shown efficacy. 40 While the relationship between SMM and cesarean delivery is interconnected and multilayered, our study showed the potential of reducing cesarean deliveries as a system-level intervention to reduce rates of SMM and associated costs. In fact, a retrospective cohort study of over 16,000 deliveries found that even after excluding cases in which vaginal delivery was contraindicated, about three-fourths of SMM cases occurred after cesarean delivery and 100% of sepsis cases were in patients with cesarean deliveries. 41 Community-level interventions include addressing social determinants of health such as housing and transportation that have established connections to health outcomes, especially amongst marginalized communities. 42 On a broader level, demonstrating differential cost distribution by region, as done in our study, can help identify region and state-level factors that contribute to SMM and are amenable to change.

Future research can focus on assessing the effectiveness of interventions in reducing both the incidence and economic burden cost of SMM. Economic studies can help identify cost-effective solutions and balance the clinical improvements with the financial investment required. Future research that looks at the full financial picture of maternal care in SMM including prenatal and postpartum care can help direct efforts toward a more proactive and comprehensive approach to caring for at-risk patients.

## Strengths and Limitations

The study's major strength pertains to the use of the largest national database for inpatient care, NIS, allowing for a large sample size. This in turn yielded results that are representative and generalizable. Our model which included patient, delivery, and hospital data allowed for a broad and comprehensive analysis of many interconnected factors.

One limitation of our study was relying on hospital charges for the delivery of hospitalization to calculate cost. This does not account for certain costs associated with hospitalization such as physician cost. In a 2022 study by Phibbs et al that looked at additional costs associated with SMM in California deliveries from 2009 to 2011, the additional cost of SMMeBTo was estimated at around $4,378 when using a model that relies on hospital cost only. This increased to $6,209 when accounting for physician fees. This shows a significant cost that is not accounted for by relying on hospital costs in our study. 4

Our study relied on the CDC's list of SMM indicators to identify deliveries with SMM which was not without flaws. The CDC's list relies less on specific diagnoses and more on associated diagnosis codes (e.g., renal failure and cardiac arrest/ventricular fibrillation) or procedure codes (e.g., hysterectomy and temporary tracheostomy) that show that a severe complication took place with the delivery. In a study that compared the CDC's definition to an expert consensus-derived clinical gold standard, the sensitivity was 0.77 and the positive predictive value was 0.44. 43 A major reason for the low positive predictive value was the high false positive rate associated with the blood transfusion procedure code (99.0 × ). This procedure code does not include a number of units transfused whereby the gold standard in the study would require transfusion of ≥4 units, in line with the definition by the Society of Maternal Fetal Medicine. 9 The issue with including blood transfusion of any amount lies in the fact that transfusion of one to two units does not necessarily correlate with severe outcomes. 43 To overcome that, our study included the category of SMMeBTo.

Furthermore, our study utilized data from 2014 to 2019, during which a transition from ICD-9 to ICD-10 coding occurred in 2015. This shift may have influenced the rates at which SMM was identified. Notably, a study by Metcalfe et al reported a statistically significant decrease in the incidence of SMM in hospital discharge data following the adoption of ICD-10 compared with ICD-9. A similar pattern is observed in our findings, with the rate of aSMM decreasing from 1.9% in 2014 and 2015 to 1.7% in 2016 and 1.8% in 2017. 44

## Conclusion

In conclusion, the cost of SMM is a crucial metric to track and analyze, as it provides insight into the large financial burden placed on the healthcare system. Although this study did not explore the underlying causes or potential solutions, examining cost variations by race, insurance type, delivery methods, and hospital region among others can help identify key disparities and highlight areas for improvement in maternal healthcare.
