# Supplementary material for: Hospital Costs of Severe Maternal Morbidity Hospitalizations in the United States from 2014 to 2019: A Nationwide Cross-Sectional Study
Source: Am J Perinatol. 2025 Jun 11;43(3):344–54. doi: 10.1055/a-2618-7331 (PMC12885625; doi:10.1055/a-2618-7331)
Supplement: Supplementary file 1 — Supplementary Material [file 10-1055-a-2618-7331-s25feb0135.pdf]

Supplementary Table S1 Center for disease control list of severe maternal morbidity indicators

Temporary tracheostomy
Ventilation
Cardiac arrest or ventricular fibrillation
Acute respiratory distress syndrome
Shock
Conversion of cardiac rhythm
Amniotic fluid embolism
Hysterectomy
Acute myocardial infarction
Sepsis
Acute renal failure
Pulmonary edema/acute heart failure
Sickle cell disease with crisis
Heart failure/arrest during surgery or procedure
Air and thrombotic embolism
Puerperal cerebrovascular disorders
Aneurysm
Blood transfusion
Disseminated intravascular coagulation
Severe anesthesia complications
Eclampsia

Supplementary Table S2 Adjusted costs with an interaction term of year and SMM

Year admitted	nSMM	aSMM	SMMeBTo
2014	$4,935.62 ($4,919.24–4,952.05)	$10,114.99 ($9,995.33–10,236.08)	$9,815.30 ($9,601.42–10,033.94)
2015	$5,050.09 ($5,033.27–5,066.97)	$10,413.84 ($10,292.38–10,536.74)	$10,490.67 ($10,257.48–10,729.16)
2016	$5,182.33 ($5,164.98–5,199.74)	$10,990.73 ($10,856.92–11,126.19)	$11,459.56 ($11,218.73–11,705.56)
2017	$5,307.47 ($5,289.64–5,325.35)	$11,517.75 ($11,380.72–11,656.43)	$12,289.00 ($12,028.12–12,555.53)
2018	$5,301.83 ($5,283.95–5,319.77)	$11,613.97 ($11,472.89–11,756.79)	$12,422.80 ($12,151.56–12,700.10)
2019	$5,541.57 ($5,522.83–5,560.38)	$12,089.47 ($11,950.58–12,229.98)	$12,872.08 ($12,612.98–13,136.51)

Supplementary Table S3 Adjusted costs with an interaction term of race and SMM

Race/ethnicity	nSMM	aSMM	SMMeBTo
Black, non-Hispanic	$5,356.41 ($5,339.05–5,373.83)	$11,370.19 ($11,252.28–11,489.34)	$12,347.07 ($12,115.38–12,583.19)
White, non-Hispanic	$5,061.78 ($5,045.36–5,078.26)	$10,465.18 ($10,381.66–10,549.38)	$10,550.99 ($10,411.19–10,692.67)
Hispanic	$5,285.76 ($5,268.63–5,302.95)	$11,298.38 ($11,180.94–11,417.06)	$12,171.11 ($11,921.95–12,425.48)
Asian or Pacific Islander, non-Hispanic	$5,976.02 ($5,953.11–5,999.00)	$13,748.75 ($13,472.29–14,030.89)	$14,685.97 ($14,123.57–15,270.78)
Native American, non-Hispanic	$5,574.49 ($5,538.03–5,611.19)	$12,028.90 ($11,432.46–12,656.45)	$13,158.20 ($11,975.42–14,457.79)
Other, non-Hispanic	$5,335.08 ($5,314.77–5,355.46)	$11,123.79 ($10,882.16–11,370.78)	$11,920.30 ($11,413.84–12,449.23)
Unknown	$5,139.08 ($5,120.16–5,158.06)	$11,401.59 ($11,134.26–11,675.35)	$12,090.52 ($11,593.07–12,609.31)

 © 2025. The Author(s).

Supplementary Table S4 Adjusted costs with an interaction term of expected primary payer and SMM

Primary payer	nSMM	aSMM	SMMeBTo
Medicaid	$5,171.48 ($5,154.98–5,188.02)	$10,923.04 ($10,843.49–11,003.18)	$11,950.55 ($11,790.57–12,112.71)
Private	$5,287.20 ($5,269.29–5,305.18)	$11,279.48 ($11,190.77–11,368.88)	$11,126.78 ($10,978.78–11,276.77)
Self-pay	$4,711.33 ($4,690.70–4,732.04)	$10,178.26 ($9,898.55–10,465.88)	$10,547.03 ($10,011.21–11,111.52)
Other	$5,130.41 ($5,109.40–5,151.51)	$11,699.30 ($11,437.02–11,967.61)	$11,893.29 ($11,519.55–12,279.15)

Supplementary Table S5 Adjusted costs with an interaction term of household income and SMM

Median household income for patient's ZIP code	nSMM	aSMM	SMMeBTo
0–25th	$4,938.51 ($4,922.59–4,954.47)	$10,370.16 ($10,282.69–10,458.38)	$11,208.92 ($11,035.74–11,384.82)
26th–50th	$5,069.56 ($5,052.78–5,086.40)	$10,815.32 ($10,709.04–10,922.65)	$11,225.71 ($11,030.17–11,424.72)
51st–75th	$5,245.47 ($5,227.76–5,263.24)	$11,498.27 ($11,376.24–11,621.62)	$11,704.17 ($11,489.01–11,923.37)
76th–100th	$5,719.01 ($5,698.90–5,739.19)	$12,433.60 ($12,289.39–12,579.50)	$12,271.47 ($12,020.82–12,527.33)

Supplementary Table S6 Adjusted costs with an interaction term of delivery method and SMM

Delivery method	nSMM	aSMM	SMMeBTo
Vaginal	$4,362.65 ($4,348.33–4,377.01)	$9,100.43 ($9,027.43–9,174.01)	$9,236.93 ($9,112.89–9,362.66)
Cesarean	$7,196.75 ($7,172.00–7,221.58)	$12,510.67 ($12,425.98–12,595.93)	$13,750.91 ($13,587.38–13,916.41)

Supplementary Table S7 Adjusted costs with an interaction term of hospital bed size and SMM

Hospital bed size	nSMM	aSMM	SMMeBTo
Small	$5,435.03 ($5,416.56–5,453.57)	$10,624.67 ($10,504.74–10,745.97)	$10,327.96 ($10,112.72–10,547.78)
Medium	$5,031.11 ($5,014.22–5,048.05)	$10,216.02 ($10,125.81–10,307.03)	$10,335.19 ($10,168.16–10,504.97)
Large	$5,252.23 ($5,234.73–5,269.79)	$11,749.84 ($11,663.52–11,836.81)	$12,485.33 ($12,332.07–12,640.49)

Supplementary Table S8 Adjusted costs with an interaction term of hospital region and SMM

Hospital region	nSMM	aSMM	SMMeBTo
Northeast	$6,095.58 ($6,074.63–6,116.61)	$11,822.40 ($11,693.44–11,952.79)	$11,993.83 ($11,748.95–12,243.81)
Midwest	$5,109.25 ($5,092.19–5,126.37)	$10,792.45 ($10,671.71–10,914.56)	$11,122.02 ($10,920.41–11,327.37)
South	$4,527.03 ($4,512.29–4,541.82)	$9,559.87 ($9,485.23–9,635.11)	$10,049.94 ($9,902.85–10,199.21)
West	$5,892.15 ($5,871.95–5,912.42)	$13,836.06 ($13,681.76–13,992.11)	$14,080.31 ($13,814.26–14,351.48)

 © 2025. The Author(s).

Supplementary Table S9 Adjusted costs with an interaction term of hospital location/teaching status and SMM

Hospital location/teaching status	nSMM	aSMM	SMMeBTo
Rural	$5,350.83 ($5,332.58–5,369.14)	$9,675.38 ($9,548.46–9,803.99)	$9,120.79 ($8,856.13–9,393.35)
Urban nonteching	$4,898.97 ($4,882.51–4,915.49)	$9,689.35 ($9,584.32–9,795.54)	$9,687.64 ($9,492.23–9,887.07)
Urban teaching	$5,306.74 ($5,289.01–5,324.53)	$11,676.98 ($11,600.63–11,753.83)	$12,236.45 ($12,105.84–12,368.48)

 © 2025. The Author(s).
